# General practitioners’ perspectives, preferences, and practices in prescribing antihypertensive medication in primary, uncomplicated hypertension

**DOI:** 10.1093/fampra/cmaf059

**Published:** 2025-08-14

**Authors:** Jakob L Schroevers, Marinho Patrick Witvliet, Eric P Moll van Charante

**Affiliations:** Department of General Practice, Amsterdam UMC, University of Amsterdam, Meibergdreef 9, 1105 AZ, Amsterdam, the Netherlands; Department of General Practice, Amsterdam UMC, University of Amsterdam, Meibergdreef 9, 1105 AZ, Amsterdam, the Netherlands; Department of General Practice, Amsterdam UMC, University of Amsterdam, Meibergdreef 9, 1105 AZ, Amsterdam, the Netherlands; Department of Public and Occupational Health, Amsterdam UMC, University of Amsterdam, Meibergdreef 9, 1105 AZ, Amsterdam, the Netherlands

**Keywords:** general practice, hypertension, antihypertensive agents, guideline, primary health care, qualitative research

## Abstract

**Background:**

International guidelines consider most antihypertensive medication (AHM) classes as equivalent options for treating primary hypertension. However, limited research has examined whether general practitioners (GPs) share this view or have specific prescribing preferences. Understanding GPs’ perspectives is crucial for identifying how guidelines are implemented in daily practice. This study explores the perspectives, preferences, and prescribing practices of Dutch GPs regarding AHM classes in patients with primary hypertension who have no cardiovascular comorbidities or diabetes.

**Methods:**

We conducted a qualitative study with semi-structured interviews among Dutch GPs. Interviews were audio-recorded, transcribed verbatim, and thematically analyzed.

**Results:**

We interviewed 18 Dutch GPs (56% female) and identified three key themes: contextual factors when initiating treatment, preferences for AHM classes, and considerations on combination therapy. GPs consider lifestyle modifications, patient age, and initial blood pressure (BP) when deciding on treatment. Most GPs do not view all AHM classes as interchangeable, with their preferences shaped by perceived efficacy, side effects, and patient-specific factors, including ethnicity and patient preferences. GPs often favour a gradual titration approach, starting with one class before adjusting the dosage or adding another.

**Conclusion:**

GPs adopt a multifaceted, patient-centred approach to hypertension, prioritizing lifestyle interventions and weighing short-term risks against long-term benefits. We identified several discrepancies between guideline recommendations and everyday practice—particularly regarding the perceived non-equivalence of AHM classes and limited support for initiating combination therapy. Incorporating GPs’ perspectives into guideline development may lead to more practical, tailored recommendations that improve adherence and patient outcomes in real-world care.

**Key messages**

1. GPs prioritize lifestyle and contextual factors in managing hypertension care.
2. Antihypertensive preferences are shaped by short-term risks and long-term benefits.
3. Incorporating GPs’ views into guidelines may improve adherence and patient outcomes.

## Background

In the pharmacological treatment of (perceived) primary, uncomplicated hypertension—defined as hypertension without an identifiable secondary cause and without diabetes or cardiovascular comorbidity—most international guidelines consider several antihypertensive medication (AHM) classes to be equivalent first-line options. In this context, e*quivalence* refers to comparable average efficacy in lowering blood pressure (BP) and reducing cardiovascular risk at the population level. These classes include angiotensin-converting enzyme (ACE) inhibitors, angiotensin receptor blockers (ARBs), (dihydropyridine) calcium channel blockers (CCBs), (thiazide) diuretics, and, to a lesser extent, beta-blockers [1–3]. Historically, diuretics and beta-blockers were the most commonly used AHM classes [4], but more recently, CCBs and renin-angiotensin system (RAS)-inhibitors, particularly ACE inhibitors, have gained popularity [5–7].

Although guidelines recommend that general practitioners (GPs) select from these equivalent classes, there is a striking lack of research on whether GPs actually perceive these medications as interchangeable—that is, whether they feel free to select any class without a specific guideline-driven rationale—or whether they hold specific preferences when managing primary, uncomplicated hypertension. Variations in prescription patterns may arise based on factors such as patient age, BP levels, or side effects associated with certain AHM classes.

Exploring clinical expertise—a cornerstone of evidence-based medicine [8]—could offer valuable insights into how GPs implement guidelines in practice, helping to explain observed prescription patterns, and potentially informing future guideline adaptations. In this study, we aimed to investigate the perspectives, preferences, and practices of Dutch GPs regarding the prescription of AHM classes for patients with primary, uncomplicated hypertension.

## Methods

### Participants

We conducted semi-structured interviews with 18 Dutch GPs [9], recruited through personal networks, snowball sampling (where interviewees recommended colleagues with differing views), and random emails to GP practices. This approach ensured a purposive sample based on sex, years of experience, practice location (urban vs. rural), and practice size.

Initially, 41 GPs received an email with study details, followed by two reminders. Of these, 15 (37%) did not respond, and 8 (20%) declined, primarily due to time constraints. The sample size was guided by data saturation, reached when consecutive interviews yielded no new insights.

### Data collection, coding, and analysis

Between February 2021 and December 2023, two physician-PhD students (JLS and MPW, both male) conducted semi-structured interviews using an interview guide (Supplementary Methods S1). Interviewers and interviewees were aware of each other’s profession. The interview guide focused on the perspectives, preferences and practices in treating newly diagnosed primary, uncomplicated hypertension, exploring specific preferences for AHM classes, and considerations on combination therapy (defined in this manuscript as the simultaneous use of two AHM classes, either as separate pills or as a fixed-dose combination tablet). At the time of the interviews, earlier versions of the hypertension guidelines—which are part of a broader Cardiovascular Risk Management Guideline—were still in effect [1, 3]. By the time of manuscript preparation, the guidelines had been updated [10, 11]. However, some of the forthcoming changes were already addressed during the interviews. The topics of AHM preferences by ethnic background and decisions on discontinuing treatment were not explored, as they were previously investigated [12, 13].

JLS and MPW conducted the first two interviews together to standardize their interviewing techniques. During data collection, they refined the interview guide to improve question clarity and outcomes. Interviews lasted 40–75 min; no repeat interviews were deemed necessary. Due to COVID-19 restrictions, only six interviews were conducted in person; the remaining twelve were remote: eight via video conferencing, and four by phone. All interviews were audio-recorded with permission and transcribed verbatim; no field notes were taken. JLS, MPW, and senior researcher EPMvC periodically reviewed findings to identify key themes.

The authors coded the interviews and conducted a thematic analysis following Braun and Clarke’s six-phase framework (Supplementary Methods S2) [14], using MaxQDA software [15]. The study was reported in accordance with COREQ guidelines (Supplementary Methods S3) [16].

## Results

We interviewed 18 GPs (Table 1), of whom 10 (56%) were female. Their experience ranged from 1 to 37 years, with an average of 15.7 years and a median of 9.5 years. Eight (44%) worked in rural areas, and 7 (39%) operated solo practices. Individual characteristics of participating GPs are detailed in Supplementary Table S1.

**Table 1.** Small practices were defined as those with one or two GPs, while larger practices consisted of three or more.

Sex (women)	10 (56%)
Years of experience as a GP	
0–15 years	11 (61%)
> 15 years	7 (39%)
Median (IQR)	10 years (7–20)
Mean (range)	14 years (1–37)
Current practice setting	
Urban	10 (56%)
Rural	8 (44%)
Current practice size	
Small	7 (39%)
Large	11 (61%)

Abbreviations: GP = general practitioner; IQR = interquartile range.

Our thematic analysis identified three main themes: (i) contextual factors when initiating treatment, (ii) AHM class preferences, and (iii) considerations on combination therapy.

### Contextual factors when initiating treatment

When managing newly diagnosed primary, uncomplicated hypertension, several contextual factors—including lifestyle behaviours, age, initial BP levels, and patient preferences—guide GPs in deciding how to initiate treatment.

#### Lifestyle modifications

GPs emphasized the importance of lifestyle modifications, particularly for younger patients, as the first-line approach to managing hypertension. They prioritized changes like dietary improvements, increased physical activity, and weight management to postpone or potentially eliminate the need for medication.

Particularly with younger patients, I tend to prioritise lifestyle changes first, as I prefer not to over-medicalise people. (GP9)

Even after initiating AHM, GPs continued to emphasize lifestyle modifications as a key component of hypertension management.

I believe lifestyle changes should always be prioritised. When blood pressure lowers, that’s when lifestyle adjustments are crucial. With effort, you might be able to reduce or even stop blood pressure medication. (GP11)

However, in older patients, maintaining lifestyle changes appeared more difficult due to the increased likelihood of physical limitations.

And the elderly, who already face reduced mobility due to osteoarthritis or other conditions, I think: yes, lifestyle interventions, at least in terms of exercise, offer fewer opportunities there. (GP11)

#### Age

GPs were generally reluctant to initiate AHM in both older and younger patients, weighing the short-term risks of side effects and the lifelong commitment to medication against the less tangible long-term cardiovascular benefits. For older adults, GPs prioritized overall vitality and activity levels over chronological age, tailoring strategies for fit and active individuals compared to those who were frail.

It’s very much a discussion with the patient, but if they’re a lively individual, you might think: this person could still have another 15 years ahead. If they’re more vulnerable, and you think lowering their blood pressure might just make them dizzy and cause a fall, then you’re doing more harm than good. (GP14)

GPs had differing views on starting AHM in younger patients. Some noted that younger individuals may lack vascular damage or comorbidities but emphasized the long-term risks of untreated hypertension.

But if someone is only 35 and has a systolic blood pressure of 165mmHg, I will treat them anyway (…) they’re still putting strain on their blood vessels for another 30 years. (GP8)

Others, however, felt that initiating AHM at a younger age requires strong justification due to the lifelong commitment to medication, stressing the need to carefully balance the potential benefits with the long-term implications.

Age is definitely important, because it’s a big step to put someone who’s, say, 40 years old on AHM—they’ll have to take it for the rest of their life. I find that quite significant. (GP9)

#### Initial BP levels

GPs’ decisions on hypertension management were strongly influenced by initial BP readings. Many viewed a systolic BP over 180 mmHg as a clear indication to start AHM immediately, bypassing lifestyle modifications.

If a young person has a systolic blood pressure consistently above 180mmHg, that’s a clear indication for treatment, even if their overall 10-year risk isn’t high. (GP6)

For lower BP values, GPs weighed whether to start AHM or delay treatment to first assess the outcomes of lifestyle modification.

#### Patient preferences

According to the interviewees, some patients preferred medication over lifestyle modifications due to concerns about cardiovascular risk, viewing it as a more immediate and reliable solution.

Yes. Some people are very clear: ‘No, just give me the tablets, I really don’t want to take this risk.’ And that’s perfectly fine. (GP8)

Others found medication more convenient than the effort required for lifestyle changes to lower BP.

But I also have people who seem disengaged [when discussing lifestyle modifications], so I’ll ask: ‘Or would you prefer a tablet?’ and they’ll say, ‘Yes, just give me that. I find it easier.’ That’s perfectly fine with me as well. It’s about the end goal. (GP7)

In some cases, rapid medication use was deemed necessary, such as before surgeries requiring controlled BP.

And sometimes it gets a bit more complicated. You hear, ‘I need surgery in a month, and my BP is far too high. It needs to come down now! Otherwise, my surgery won’t go ahead.’ In such situations, it’s sometimes necessary to prescribe medication straight away. (GP18)

Conversely, patients opted to delay medication, focusing on lifestyle changes first, with GPs offering guidance or referrals to specialists like dietitians.

“People tell me: ‘I want to try it myself first.’ And that’s fine; I’ll at least direct them to [the national GP-run patient information website] and say: ‘Have a read through that and see what you can do about your lifestyle.’ I also offer to involve a dietitian if they are overweight. (GP13)

### AHM class preferences

Only a few GPs agreed that the five main AHM classes were truly interchangeable options for initiating treatment in newly diagnosed primary, uncomplicated hypertension.

Yes, in principle. It varies from person to person, but that’s true for nearly all medications, really. (GP8)

However, most interviewed GPs had specific preferences or avoided certain classes in particular situations. Several GPs acknowledged that aspects of earlier guidelines—such as the previously prominent position of diuretics—still influenced their current prescribing practices.

I still have a tendency to prescribe diuretics. (GP1)

Many preferred starting with ACE inhibitors, especially for younger patients without comorbidities, mentioning their nephroprotective properties and manageable side effects. ARBs were generally reserved for patients who experienced side effects from ACE inhibitors. CCBs were favoured for patients with higher initial BP readings, as they were perceived to be faster-acting and more potent. Additionally, GPs also preferred CCBs for patients of African descent. In other cases, CCBs were generally avoided as a first choice due to side effects like oedema. Diuretics, once a go-to option, were typically added as a second-line treatment or reserved for milder hypertension. Their decline in popularity was tied to concerns about skin cancer and dehydration during hotter summers, particularly in older individuals. Beta-blockers, which were the second most popular class two decades ago, were no longer a first choice for any of the interviewed GPs. The main reasons were their perceived limited antihypertensive effect and side effects, particularly in younger patients who experienced lower exercise tolerance. However, beta-blockers were still occasionally preferred when hypertension is accompanied by anxiety or palpitations.

Some GPs appreciated the guideline recommendations that offered greater flexibility in choosing an antihypertensive class for patients with primary, uncomplicated hypertension, while others expressed a desire for more explicit guidance.

I feel that if there’s no clear-cut preference, it gives me the freedom to handle it in my own way—which I really appreciate. (GP12)

I would actually welcome a bit more guidance, even though I used to regularly deviate from the old guidelines. (GP11)

All other quotes related to this theme are presented in Table 2, categorized by AHM class.

**Table 2.** General practitioners' quotes on AHM class characteristics.

	AHM classes			
	RAS inhibitors	Calcium channel blockers	Diuretics	Beta-blockers
1.Specific beneficial effects	‘You increasingly hear that ACE inhibitors are better in the long run for additional benefits, such as protecting kidney function and improving cardiovascular outcomes.’-**GP12**	‘So, if you have very high blood pressure that needs to be lowered quickly, in my experience, a calcium channel blocker often works best.’**-GP3**	‘And if we’re talking about someone who only needs a small amount, I might start with hydrochlorothiazide.’**-GP7**	‘Yes, but mainly a beta-blocker, and only occasionally for an 85-year-old woman who’s extremely anxious, which is causing high blood pressure.’**-GP6**
2. Side effects	‘Previously, I used ACE inhibitors more frequently, but I’ve noticed how often people experience coughing issues, so I end up switching to ARBs quite regularly.’**-GP4**	‘No, I think the reason for using calcium channel blockers later on is indeed because we often see peripheral oedema.’**-GP2** ‘With amlodipine, the side effect profile is broader, which makes it less straightforward to advise patients and somewhat harder to manage.’**-GP3**	‘And especially now, with the changing climate and much warmer summers (...) diuretics used to be the first choice, but I find them less favourable for elderly patients, particularly with the risk of dehydration.’**-GP16**	‘It used to be the first choice, but many people actually experience issues with the reduced heart rate, particularly those who are still active—they struggle with physical activities. It’s also not ideal for older adults due to the slow pulse and an increased risk of falling. So, unless a patient has arrhythmias, I generally wouldn’t prescribe beta-blockers anymore.’**-GP16**
3. Other considerations	‘ARBs, yes, I prescribe them to people—for instance, those with migraines or who experience side effects from other antihypertensive medication classes.’**-GP16**	‘And I prefer prescribing CCBs to Creole and African patients. They’re generally more effective than diuretics, which are regarded as equivalent in the guidelines for these ethnic groups.’**-GP10**	‘There was that issue with hydrochlorothiazide and basal cell carcinomas a while back – that’s something I keep in mind. These are medications people have to take for their entire lives.’**-GP12**	‘(…) it also causes more side effects, and I just want to encourage people to exercise, participate in sports, and so on.’**-GP9** ‘Definitely not with the beta-blocker; they’re less effective.’**-GP16**

Abbreviations: Quotes are organised by specific beneficial effects, side effects, and other considerations across the different AHM classes. ACE = angiotensin-converting enzyme; AHM = antihypertensive medication; ARB = angiotensin receptor blocker; CCB = calcium channel blocker; GP = general practitioner; RAS = renin–angiotensin system.

### Considerations on combination therapy

Most GPs found that, with some persuasion, patients were generally willing to accept adding a second AHM class rather than increasing the dosage when hypertension was not yet adequately controlled.

I don’t leave much room for discussion in that regard. A combination [of two classes] works much better for me than simply increasing the dosage of one. And if you explain to them that it’s scientifically proven that multiple medications are more effective than increasing the dosage, they generally accept this approach. (GP3)

However, some acknowledged that persuading patients to take an additional medication can sometimes be challenging.

But it is often harder to motivate people; they prefer to go from Lisinopril 10 to 20mg rather than take an additional tablet, and I can see why they feel that way. (GP2)

When asked whether they routinely followed the guideline recommendation to add a second antihypertensive class—rather than increasing the dose of the initial drug—when BP introduced another class, either due to their own preference or that of the patient. This was often because some GPs started with a marginally effective dosage to assess tolerance, opting to increase the dose before considering a second class.

Well, it depends a bit. Often, we start by increasing the dose and then add something. Monotherapy is often more acceptable to the patient than starting multiple medications at once. To be honest, I wouldn’t want to take two tablets every day either if I could manage with just one. (GP15)

One of the upcoming changes in the Dutch hypertension guideline at the time was the recommendation to initiate dual-class antihypertensive therapy in patients with elevated initial BP values (≥ 150 mmHg) [10]. When discussing this proposed update, GPs generally acknowledged that a single AHM class would likely be insufficient in cases of very high initial BP. However, only a few stated that they already initiated or intended to initiate treatment with two classes simultaneously. Most expressed a preference to start with one class and then, after a few weeks, evaluate the need for a second, aligning well with the continuity of care in primary care settings.

I think few GPs are accustomed to initiating two classes at the same time; at least, I don’t see this happening any time soon in my workplace. (GP17)

No, I’ve never done that. If the blood pressure is very high and doesn’t decrease with the first antihypertensive medication after a week to ten days, then I might quickly add a second one. (GP8)

Concerns about a rapid BP decline causing more side effects, potentially negatively affecting adherence, also influenced this approach.

If you prescribe too many AHM, someone might collapse, and then treatment adherence is completely lost. I’ll have them return a bit sooner. (GP16)

Additionally, starting with one class makes it easier to identify side effects.

No, I actually never do that. Because if there are side effects, you won’t know exactly which medication is causing them. (GP17)

Still, in some situations, some GPs did already initiate treatment with two classes at once.

Well, if I already have the impression that one AHM won’t be enough, then very occasionally I do prescribe a fixed combination tablet and tell them to take half for the first two weeks. If all goes well, then they take the full tablet. (GP10)

## Discussion

This study explored how Dutch GPs manage newly diagnosed primary, uncomplicated hypertension, identifying three key themes: contextual factors when initiating treatment, AHM class preferences, and considerations on combination therapy.

Contextual factors when initiating treatment included lifestyle modifications, patient age, initial BP, and patient preferences. GPs prioritized lifestyle changes, particularly for younger patients, to avoid early medicalization, balancing short-term side effects with long-term benefits. Contrary to the guideline’s stance that AHM classes are largely equivalent for treating primary hypertension, most GPs in our study did not consider them interchangeable. Their preferences were shaped by perceived efficacy, side effects, patient-specific characteristics, and earlier guideline recommendations.

Likewise, the proposed recommendation to initiate treatment with two AHM classes simultaneously—a pending guideline change at the time of the interviews—was met with limited enthusiasm. This raises questions about how easily such recommendations will be adopted in practice, although attitudes may shift over time. Interviewed GPs often preferred a stepped approach, starting with monotherapy to better identify side effects and promote shared decision-making. They also voiced concerns that a sudden BP drop initiating dual therapy could undermine patient adherence. These findings are consistent with a 2007 phone survey among GPs, that found a similar preference for monotherapy as initial treatment [17]. Rather than dismissing these concerns, acknowledging them in the guideline—with room for initial monotherapy followed by timely escalation—may support a more effective uptake of recommendations while still promoting evidence-based care.

Interviewed GPs generally preferred ACE inhibitors for younger patients. Although this was not explicitly confirmed in the data, their choices may reflect the influence of earlier guidelines—particularly the AB/CD rule, which recommended ACE inhibitors or beta-blockers for younger, non-black patients, and calcium channel blockers or diuretics for older or black patients. ARBs were mainly prescribed for patients intolerant to ACE inhibitors, likely due to their more recent introduction and less established role in practice [18]. We expected to find other prescribing patterns, such as increased use of thiazide diuretics and beta-blockers among GPs trained under earlier guidelines, but no consistent trends were observed. Consistent with guidelines, CCBs were preferred for patients of African ancestry. They were also commonly used in patients with higher initial BP, which is not supported by current guidelines. Meanwhile, thiazide diuretics and beta-blockers have declined in popularity as first-line options.

This decreased popularity aligns with data confirming a decline in the use of diuretics and beta-blockers [5–7, 19]. Several GPs attributed their shift away from thiazide diuretics to concerns about the link between these drugs—particularly hydrochlorothiazide—and an increased risk of skin cancer [20, 21], prompting regulatory agencies, including the FDA, to issue warnings [22–24]. In response, the Netherlands saw a 45% reduction in hydrochlorothiazide prescriptions the following year [25]. Meanwhile, beta-blockers were almost entirely avoided due to perceived limited effectiveness and negative impact on exercise tolerance. Although beta-blockers were still a first-line option in prevailing guidelines [1, 3], concerns have recently emerged about their clinical inferiority and burdensome side effects [11, 26], concerns echoed by the GPs in this study. RAS inhibitors have gained popularity over the last decades, reflected by increasing prevalence [27]. However, GPs did not provide a clear rationale for preferring ACE inhibitors over ARBs; factors like longer availability, stronger evidence, and, until recently, lower cost likely influenced this preference [28]. Regarding CCBs, several interviewees perceived them as more effective, achieving greater or faster BP reductions compared to other classes. Although CCBs are commonly used in hypertensive emergencies, there is no clear evidence that they outperform other classes in the treatment of primary hypertension [1–3].

Physicians’ views and experiences are important for understanding the challenges GPs face in everyday hypertension management. Evidence indicates that adherence to updated hypertension guidelines remains suboptimal, highlighting the need for qualitative studies to uncover the underlying reasons [29–31]. Recently, the European Society of Cardiology launched an initiative to improve guideline adoption in primary care, further emphasizing the importance of understanding hypertension management practices in this context [32]. Nonetheless, there remains a notable lack of qualitative research exploring the perspectives, preferences, and practices of GPs and other treating physicians on hypertension management.

### Strengths and limitations

To our knowledge, this is the first qualitative study to explore GPs’ insights when prescribing AHM for primary, uncomplicated hypertension. Understanding their decision-making processes can offer valuable insights into the application of clinical guidelines in practice, potentially guiding future adaptations to enhance their relevance and usability.

Despite the challenge of capturing a full range of perspectives in qualitative research, we believe our interviews included a diverse sample of Dutch GPs with a variety of backgrounds, practice types, and levels of experience.

Some potential limitations need consideration when interpreting our results. Several interviews were conducted via telephone due to physician preferences and COVID-19 restrictions, potentially limiting non-verbal communication. However, we believe this impact was minimal, as the quality and openness of these audio-only interviews were comparable to those conducted in person or via video. With less than half of the invited GP participating, responder bias may have been introduced, as those more interested in hypertension management may have been more likely to respond. Finally, this study focused on GPs management of newly diagnosed hypertension, while follow-up care is often handled by practice nurses. Future studies incorporating nurses’ perspectives could provide a more comprehensive understanding of hypertension management in primary care.

## Conclusion

GPs navigate a multifaceted, patient-centred decision-making process in managing primary, uncomplicated hypertension, prioritizing lifestyle interventions while balancing short-term risks—such as medicalization, potential side effects, and non-adherence—against long-term cardiovascular benefits. This study identified several discrepancies between clinical practice and guideline recommendations, particularly regarding the perceived non-equivalence of AHM classes and the reluctance to initiate hypertension treatment with combination therapy, which GPs neither supported nor consistently followed. Acknowledging healthcare professionals’ perspectives in updating hypertension guidelines may enhance adherence while still promoting new evidence. Further qualitative research, for instance further zooming in on GP’s views on combination therapy, could help bridge the gap between guidelines and real-world practice, leading to more tailored recommendations that enhance adherence and ultimately improve patient outcomes.

## Supplementary Material

cmaf059_suppl_Supplementary_Tables_1

## Data Availability

The data underlying this article cannot be shared publicly due to participant privacy, but requests for research purposes will be considered upon reasonable inquiry.
