# Supplementary material for: General practitioners’ perspectives, preferences, and practices in prescribing antihypertensive medication in primary, uncomplicated hypertension
Source: Fam Pract. 2025 Aug 14;42(5):cmaf059. doi: 10.1093/fampra/cmaf059 (PMC12351541; doi:10.1093/fampra/cmaf059)
Supplement: cmaf059_suppl_Supplementary_Tables_1 [file cmaf059_suppl_supplementary_tables_1.docx]

**Online supplemental content to**

“General Practitioners’ Perspectives, Preferences, and Practices in Prescribing Antihypertensive Medication in Primary, Uncomplicated Hypertension”

**Supplementary Methods 1.** Interview guide.

**Part I: Background**

To gain a comprehensive understanding of your work experience and approach for this study, I'd like to begin by asking you some questions about yourself and your practice.

- Could you tell me a bit about yourself and your background as a general practitioner?
  - (Years of experience, type of practice—urban or rural, solo or group practice, experience prior to GP training, additional specializations, socioeconomic status and composition of your neighbourhood)
- What does hypertension management look like in your practice? (Do you have a practice nurse who handles this, or do you manage it yourself?)
  - How do you and the practice nurse divide responsibilities in hypertension care?
- How do you approach the prevention of cardiovascular diseases in your practice?
  - Do you invite all patients above a certain age for screening?
  - Do you assess cardiovascular risk when patients visit for other reasons?
- What role does lifestyle intervention play in hypertension management within your practice?

**Part II: Main Section**

I'd now like to ask you some questions about antihypertensive medications, starting with how you choose them.

- Can you describe your approach when prescribing antihypertensive medication to a patient for the first time?
- What are your thoughts on the section of the hypertension guideline regarding antihypertensives?
  - Is it too detailed or specific (more information or recommendations than necessary)?
  - Is it not detailed or specific enough (are there recommendations or guidance you feel are missing)?
  - Is it clear when certain recommendations apply and when they do not?

In recent years, the hypertension guideline has stated that various groups of antihypertensives—ACE inhibitors, ARBs, beta-blockers, calcium channel blockers, and diuretics—are considered equivalent options for people with hypertension without comorbidities, providing similar blood pressure-lowering effects.

- Do you find this equivalence holds true in your experience, or do you feel certain medications work better?
- Do you have a preference for a particular group of antihypertensives or a specific medication?
  - Clarification: When you initiate antihypertensive therapy, which group or medication do you typically choose?
  - Clarification 2: How do side effects influence your choice of antihypertensive?
  - Clarification 3: If certain medication classes (ACE inhibitors, ARBs, beta-blockers, calcium channel blockers, diuretics) aren't mentioned, could you explain why?
  - Clarification 4: If you prefer ACE inhibitors over ARBs, why do you choose them despite the risk of a dry cough, a side effect not associated with ARBs?
- How did your preference or order of choice develop?
  - Clarification: How fixed is this order for you? If new evidence showed that a particular class of antihypertensives had a significant advantage for a specific organ system (beyond blood pressure reduction and cardiovascular event impact), would that influence your preferences?
- Are there specific patient groups—for example, ethnic minorities, patients with diabetes, or older adults—for whom you prefer a particular drug or drug class? Could you describe different patient scenarios where you'd choose different antihypertensive classes?
  - If you choose a beta-blocker for a nervous patient, is this initiated as monotherapy? If the beta-blocker doesn't sufficiently lower blood pressure, what is your next step in treatment?
- Does age play a role in your decision to prescribe antihypertensives, and if so, how?
  - Clarification: For a 45-year-old with newly diagnosed hypertension, would you start medication immediately or begin with lifestyle interventions? How about for a 65-year-old? What about a frail 75-year-old?
- How does the level of blood pressure influence your management of newly diagnosed hypertension?
  - Clarification: Would you choose a different medication for a patient with a blood pressure of 145 mmHg compared to one with 170 mmHg?
- Do you sometimes start treatment with two antihypertensives simultaneously?
  - Clarification: If not, why? Research indicates that starting with two medications can improve both mortality and morbidity compared to starting with one.

The upcoming [*National General Practitioners’*] hypertension guideline is expected to recommend initiating treatment with two medications for systolic blood pressure over 150 mmHg.

- What are your thoughts on this forthcoming recommendation?
  - Clarification: How might this influence your prescribing habits?
- Are you concerned about losing patients to follow-up, and would it be better to start directly with two antihypertensive medications?
  - Example: If a patient starts on one medication but doesn't return for follow-up, their blood pressure may remain poorly controlled. Wouldn't starting with two medications from the outset improve outcomes?

**Supplementary Methods 2.** Braun and Clarke’s framework

1. Familiarization, the interviewers (JLS & MPW) listened to audio recordings and read transcripts to familiarize themselves with the data;
2. Initial coding, both interviewers independently generated initial coding trees using MaxQDA13;
3. Theme Development, a coding system was developed to generate a thematic map of main themes and subthemes;
4. 3.Review and Refinement, codes were reviewed and refined by the interviewers to reach consensus, with input from the research group (JLS, MPW & EPMvC);
5. Narrative Development, the themes were further refined and discussed to create a narrative of the main findings;
6. Reporting, themes were used to produce the final report, with illustrative quotes translated from Dutch to English.

**Supplementary Methods 3.** Consolidated criteria for reporting qualitative studies (COREQ): 32-item checklist.

| **No. Item** | **Guide questions/description** | **Reported on Page #** |
| --- | --- | --- |
| **Domain 1: Research team and reﬂexivity** |  |  |
| *Personal Characteristics* |  |  |
| 1. Inter viewer/facilitator | Which author/s conducted the interview or focus group? | 4 |
| 2. Credentials | What were the researcher’s credentials? E.g. PhD, MD | 1, 4 |
| 3. Occupation | What was their occupation at the time of the study? | 4 |
| 4. Gender | Was the researcher male or female? | 4 |
| 5. Experience and training | What experience or training did the researcher have? | 4 |
| *Relationship with participants* |  |  |
| 6. Relationship established | Was a relationship established prior to study commencement? |  |
| 7. Participant knowledge of the interviewer | What did the participants know about the researcher? e.g. personal goals, reasons for doing the research | 4 |
| 8. Interviewer characteristics | What characteristics were reported about the inter viewer/facilitator? e.g. Bias, assumptions, reasons and interests in the research topic | N/A |

| **Domain 2: study design** |  |  |
| --- | --- | --- |
| *Theoretical framework* |  |  |
| 9. Methodological orientation and Theory | What methodological orientation was stated to underpin the study? e.g. grounded theory, discourse analysis, ethnography, phenomenology, content analysis | 5, supplementary methods 2 |
| *Participant selection* |  |  |
| 10. Sampling | How were participants selected? e.g. purposive, convenience, consecutive, snowball | 4 |
| 11. Method of approach | How were participants approached? e.g. face-to-face, telephone, mail, email | 4 |
| 12. Sample size | How many participants were in the study? | 4 |
| 13. Non-participation | How many people refused to participate or dropped out? Reasons? | 4 |
| *Setting* |  |  |
| 14. Setting of data collection | Where was the data collected? e.g. home, clinic, workplace | 4 |
| 15. Presence of non-participants | Was anyone else present besides the participants and researchers? | 4 |
| 16. Description of sample | What are the important characteristics of the sample? e.g. demographic data, date | 6, Table 1, Supplementary Table 1. |
| *Data collection* |  |  |
| 17. Interview guide | Were questions, prompts, guides provided by the authors? Was it pilot tested? | 4, Supplementary Methods 1. |
| 18. Repeat interviews | Were repeat inter views carried out? If yes, how many? | 4 |
| 19. Audio/visual recording | Did the research use audio or visual recording to collect the data? | 4 |
| 20. Field notes | Were ﬁeld notes made during and/or after the interview or focus group? | 5 |
| 21. Duration | What was the duration of the inter views or focus group? | 4 |
| 22. Data saturation | Was data saturation discussed? | 4 |
| 23. Transcripts returned | Were transcripts returned to participants for comment and/or correction? | N/A |
| **Domain 3: analysis and ﬁndings** |  |  |
| *Data analysis* |  |  |
| 24. Number of data coders | How many data coders coded the data? | Supplementary Methods 2. |
| 25. Description of the coding tree | Did authors provide a description of the coding tree? | Supplementary Methods 2. |
| 26. Derivation of themes | Were themes identiﬁed in advance or derived from the data? | 5, Supplementary Methods 2. |
| 27. Software | What software, if applicable, was used to manage the data? | 5 |
| 28. Participant checking | Did participants provide feedback on the ﬁndings? | N/A |
| *Reporting* |  |  |
| 29. Quotations presented | Were participant quotations presented to illustrate the themes/ﬁndings? Was each quotation identiﬁed? e.g. participant number? | 6-11, Table 2. |
| 30. Data and ﬁndings consistent | Was there consistency between the data presented and the ﬁndings? | 12-15 |
| 31. Clarity of major themes | Were major themes clearly presented in the ﬁndings? | 6,12 |
| 32. Clarity of minor themes | Is there a description of diverse cases or discussion of minor themes? | 6-11 |

**Supplementary Table 1.** Individual General Practitioners’ Characteristics

| General Practitioner | Sex | Years of Experience | Practice setting | Practice size |
| --- | --- | --- | --- | --- |
| 1 | Male | 27 | Rural | Small |
| 2 | Female | 7 | Urban | Small |
| 3 | Female | 10 | Urban | Large |
| 4 | Male | 1 | Urban | Large |
| 5 | Female | 1 | Urban | Large |
| 6 | Female | 5 | Rural | Small |
| 7 | Female | 7 | Urban | Small |
| 8 | Female | 10 | Rural | Small |
| 9 | Male | 20 | Rural | Small |
| 10 | Male | 17 | Urban | Large |
| 11 | Male | 8 | Rural | Large |
| 12 | Male | 19 | Urban | Large |
| 13 | Male | 37 | Rural | Large |
| 14 | Female | 4 | Rural | Large |
| 15 | Female | 9 | Urban | Large |
| 16 | Male | 25 | Urban | Large |
| 17 | Female | 8 | Urban | Large |
| 18 | Female | 27 | Rural | Small |
